# Assessment of Blood Pressure Control Among Hypertensive Patients Attending Primary Healthcare Settings in Qatar: A Retrospective Chart Review

**DOI:** 10.2147/VHRM.S573765

**Published:** 2026-06-16

**Authors:** Nada Abdelkader, Ahmed Awaisu, Hazem Elewa, Nadin Hany Kamel, Samya Ahmad Al Abdulla, Ziyad Mahfoud, Maguy Saffouh El Hajj

**Affiliations:** 1 Clinical Pharmacy and Practice Department, College of Pharmacy, QU Health, Qatar University, Doha, Qatar; 2 Primary Health Care Corporation, Doha, Qatar; 3 Weill Cornell Medicine, Qatar, Doha, Qatar

**Keywords:** blood pressure, blood pressure control, primary care, factor, predictor

## Abstract

**Aim:**

Uncontrolled blood pressure (BP) poses a significant threat to public health, contributing to increased all-cause and cardiovascular disease (CVD) mortality.

The study objectives were to assess BP control among hypertensive patients attending primary care centers in Qatar and to explore the association between patient-specific factors and BP control.

**Methods:**

A retrospective chart review from 2017 to 2020 was conducted across Qatar primary care centers. A sample size of 400 patients per year was chosen to be able to estimate the yearly proportion of patients achieving BP control (the primary outcome) to within a margin of error of at most 5% using 95% confidence interval. Univariable and multivariable logistic regression models were used to assess the relationship between patient variables and BP control.

**Results:**

Among the 2,185 selected patients, over 60% of general hypertensive patients achieved BP control, (n=1226/1859, 66% in 2018), (n=1398/2038, 69% in 2019), and (n=1229/1897, 65% in 2020). More than 60% of diabetics or patients with chronic kidney disease (CKD) reached target BP levels (n=882/1309, 67.4% in 2018), (n=1002/1402, 71.5% in 2019), and (n=878/1314, 66.8% in 2020). Gender, nationality (Qatari vs. non-Qatari), and presence of comorbidities like dyslipidemia, congestive heart failure, peripheral vascular disease, atrial fibrillation and CKD were found to significantly impact BP levels.

**Conclusion:**

This study suggests that over half of hypertensive patients in Qatar’s primary care setting achieved controlled BP. Key predictors of BP control included Qatari nationality, dyslipidemia, and CHF. However, due to the inherent limitations of retrospective chart review methodology, these results should be interpreted judiciously. Future prospective studies are crucial to corroborate these predictors and inform the development of tailored strategies to overcome barriers for achieving optimal BP control.

## Introduction

Hypertension is the leading cause of mortality, accounting for the deaths of over one billion individuals globally. 1 Additionally, it is a major risk factor for cardiovascular diseases (CVDs), increasing the risk of serious complications such as stroke, heart failure, atrial fibrillation, and other related conditions.^2^

The risk of CVDs doubles with every 20/10 mm Hg increase in blood pressure (BP) above 115/75 mm Hg.^3^ In 2021, the World Health Organization (WHO) reported that only 21% of adults diagnosed with hypertension worldwide have their BP under control.^4^ The 2019 Global Burden of Disease Study highlighted that high BP was responsible for approximately 10 million deaths in low- and middle-income countries, where only 10% of patients had their BP under control.^1^ This problem is also observed in high-income countries despite their developed healthcare systems.^5^ A cross-sectional study conducted in the State of Qatar in 2004 found that 32% of the Qatari population suffers from hypertension.^6^ Additionally, the Qatar Public Health Strategy 2017–2022 reported that in 2015, high blood pressure was among the top three risk factors responsible for mortality in the country.^7^ This trend was similarly observed in other countries such as Oman, Kuwait, and the broader Middle East and North Africa (MENA) region.^7^

It is widely recognized that effective blood pressure control is one of the most crucial strategies for reducing the CVD risk associated with hypertension^8^ and minimizing damage to target organs.^3^,^8^,^9^ The beneficial impact of antihypertensive medications in lowering CVD risk among hypertensive patients is well-documented through findings from large randomized controlled trials (RCTs).^10–13^ Additionally, a meta-analysis of RCTs conducted in 2022 demonstrated that antihypertensives effectively lower blood pressure, with the maximum effect observed 12 months after initiation.^14^ The review concluded that antihypertensives remain effective in reducing blood pressure for up to four years, emphasizing the importance of appropriate therapy regimens for sustaining long-term blood pressure control.^14^

A systematic review conducted in 2021, covering 17 MENA countries, found that only 19% of individuals had their blood pressure (BP) under control.^15^ Similarly, a systematic assessment in 2020, encompassing 22 Arab countries, reported that less than one-third of patients with hypertension achieved BP control defined as systolic blood pressure less than 140 mmHg and diastolic blood pressure less than 90 mmHg.^16^

Several factors hinder patients from achieving BP control.^17^ Key factors include patient demographics, socioeconomic status, cultural background, education, and language, all of which significantly impact BP control.^18^ Additionally, a lack of patient knowledge about the disease and its management, coupled with low adherence to medications, can further contribute to suboptimal BP control.^19^ Similarly, physicians’ knowledge, adherence to guidelines, and their approach to BP management can also act as barriers.^18^,^19^ Moreover, healthcare costs, especially those associated with hypertension screening and management, pose a significant challenge, particularly in low socioeconomic countries.^20^ Despite that behavioral and lifestyle factors such as dietary composition, exercise, alcohol consumption and tobacco use may also affect blood pressure control,^21^,^22^ these factors were not captured in the retrospective chart review and are beyond the scope of the study. In the State of Qatar, the Primary Health Care Corporation (PHCC) is the principal outpatient healthcare provider in Qatar, offering many services for managing hypertension.^23^,^24^ As the first point of contact for patients with chronic conditions such as hypertension, dyslipidemia, and diabetes mellitus, primary care has an essential role in the country in supporting hypertension management and achieving blood pressure control. Although several guidelines are available to guide the proper management of hypertension in Qatar,^25^ there is limited knowledge about the status of BP control among hypertensive adults attending PHCC centers. While a recent retrospective observational study described prescribing trends and patterns for antihypertensive agents in primary healthcare settings in Qatar,^26^ the factors associated with BP control in this setting remain poorly characterized. Therefore, the study objectives were to assess BP control among hypertensive patients attending PHCC centers and to explore the association between patient-specific factors and BP control. The ultimate goal of this study is to contribute to the improvement of hypertension management in the country in the future.^27^

## Methods

### Study Setting and Design

This retrospective study used a chart/medical record review conducted across all PHCC centers in Qatar. PHCC operates a total of 31 centers across the country.^23^ As of 2021, 141,263 individuals were registered in the PHCC database.^23^,^28^ A retrospective study design was considered the most suitable method as it enables the data collection from a large population, facilitating a thorough investigation of the study’s objectives and allowing for the analysis of effects on specific subgroups.^29^ Additionally, it benefits from collecting data over extended observation periods more conveniently and at a lower cost.^29^

### Study Participants

The inclusion criteria for study participation required patients to be over 18 years of age and to have been diagnosed with hypertension between January 2017 and March 2021. The International Classification of Diseases (ICD) 11 coding for essential hypertension was used for identification.^30^

A simple random sampling of hypertensive adult patients from the medical records of all PHCC centers in Qatar was performed using the Cerner© database, without any direct patient involvement in the study. An Excel sheet was extracted containing the selected patients’ sociodemographic data (such as age, gender, and nationality), medication history, medical information (including conditions like diabetes, congestive heart failure, stroke, chronic kidney disease, and coronary artery disease), date of hypertension diagnosis, details of prescribed antihypertensives (including route of administration and dosage regimen), past and latest blood pressure (BP) measurements, and social history (including tobacco use).

Patients were excluded from the study if they were prescribed antihypertensives for reasons other than hypertension, had secondary hypertension, or had pregnancy-induced hypertension.

### Sample Size

Approximately 400 patients were selected each year for the years 2017, 2018, 2019, and 2020. With a sample size of 400 patients per year, we were able to estimate the yearly percentage of hypertensive patients with controlled blood pressure to within a 5.0% margin of error and a 95.0% confidence interval, assuming a 50.0% prevalence. Moreover, with 2000 patients, the study sample was considered adequate to support fitting a multivariable logistic regression model for predicting blood pressure control with at least 10 independent variables assuming the commonly cited rule of about 10 events per predictor.

### Outcome Measures

This study’s primary outcome of interest is the percentage of patients achieving BP control. The cut-off points for controlled and uncontrolled systolic blood pressure (SBP) and diastolic blood pressure (DBP) values are outlined according to the PHCC hypertension management guidelines, as illustrated in Table 1. These points are based on the patient’s age, comorbidities, and other characteristics. The study’s secondary outcome is the association between BP control and various predictors, including patient demographics, comorbidities, and the number of medications (monotherapy vs. polytherapy). Data from 2017 to 2019 were assessed according to the 2016 PHCC hypertension guidelines, while the 2020 data were evaluated using the 2020 PHCC hypertension guidelines. A complete-case analysis was performed; no imputation of missing data was undertaken.

**Table 1:** PHCC Hypertension Management Guidelines

Guideline Used	Population	Blood Pressure Goals
PHCC (2016) guideline^31^ is adopted from JNC8 (2014) guideline^32^	<60 years hypertensive patients without diabetes or CKD	<150/90 mmHg
	≥ 60 years hypertensive patients without diabetes or CKD	<140/90mmHg
	Diabetic and CKD population	<140/90mmHg
PHCC (2020)^33^ adopted from NICE guideline (2019)^34^	≥ 80 years regardless of their comorbidities	<150/90mmHg
	<80 years regardless of their comorbidities	<140/90mmHg

**Abbreviations**: PHCC, Primary Health Care Corporation; JNC8, Eighth Joint National Committee; NICE, National Institute for Health and Care Excellence; CKD, Chronic Kidney Disease.

### Data Analysis

Data was analyzed using IBM SPSS (SPSS^®^ for MacOS version 29.0 (IBM Corp, Armonk, New York, USA). Continuous variables (eg.: age, SBP, and DBP) were tested for normality using the Kolmogorov–Smirnov and Shapiro–Wilk tests. Normally distributed continuous variables were summarized using means and standard deviations. Not normally distributed variables were synthesized using median and interquartile range (IQR). Categorical variables were presented through frequencies and percentages. Univariable and multivariable logistic regression models were used to assess the relationship between the independent variables (patients’ demographics, comorbidities, and medications) on the dependent variable (BP control). For the analysis, the average of the last three annual blood pressure readings was calculated.

Three multivariable models were created. The first model included all the demographic and comorbidities variables that were tested at the bivariate level. Those variables were chosen for either being significant at the bivariate level or having potential confounding effect on other variables in the model. The second multivariable model was similar to the first but also included the last therapy, categorized as either monotherapy or multiple (dual, triple, quadruple, etc.) drug therapy. The third model was similar to the second one but focused on patients who had their last therapy in 2020. Odds ratios (ORs) and adjusted ORs with 95% confidence intervals (CIs) were calculated to test the possible association between the independent and dependent variables. Potential collinearity between the covariates were assessed by computing the Variance Inflation Factors. No evidence of multicollinearity was observed (all variance inflation factors < 2). A P-value ≤ 0.05 was considered significant.

### Ethical Considerations

Ethical approval for conducting the study was obtained from the research department at the Primary Health Care Corporation (PHCC) [Ref No. PHCC/DCR/2020/06/066] and Qatar University Institutional Review Board (IRB) [Ref No. QU-IRB 1458-E/21].

Patient consent was unnecessary due to the absence of direct patient involvement.

The study complies with the Declaration of Helsinki.

## Results

### Sociodemographic Characteristics of Patients with Hypertension

A total of 2,185 medical charts were randomly selected from the Cerner^®^ database: 547 patients diagnosed with hypertension in 2017, 544 patients in 2018, 545 patients in 2019, and 549 patients in 2020. The detailed sociodemographic characteristics of patients are summarized in Table 2. There was an almost equal distribution of genders, with females accounting for 53.1% (n = 1,160). Nearly half of the patients (n=1077, 49.3%) were Qatari nationals, while the remainder represented a variety of other nationalities. The most common comorbidities were diabetes (n=1428, 65.4%), and dyslipidemia (n=1265, 57.9%).

**Table 2:** Sociodemographic Characteristics of Patients

Characteristic	Frequency (Percent)
**Age (N=2185)**	
Mean (SD)	57.91 (12.32)
Range	19- 97
**Gender (N=2185)**	
Male	1025 (46.9%)
Female	1160(53.1%)
**Country of Origin (N=2185)**	
Qatar	1077(49.3%)
India	199 (9.1%)
Egypt	148 (6.8%)
Pakistan	117(5.4%)
Philippines	83 (3.8%)
Sudan	78 (3.6%)
Jordan	73 (3.3%)
Palestine	70 (3.2%)
Bangladesh	66 (3.0%)
Syria	39 (1.8%)
Other countries^a^	235(10.7%)
**Comorbidities (N= 2185)**	
Diabetes	1428 (65.4%)
Dyslipidemia	1265(57.9%)
CKD	218(10.0%)
CHF	113(5.2%)
CVDs	106(4.9%)
AFib	82(3.8%)
Other Arrhythmias	30(1.4%)
PVD	21(1.0%)
**Attending Smoking Clinic (Yes)**	24 (1.1%)
**Date of Hypertension Diagnosis (N= 2185)**	
2017	547(25.0%)
2018	544(24.9%)
2019	545(24.9%)
2020	549(25.1%)

**Notes**: ^a^Afghanistan, Algeria, Australia, Bahrain, Canada, Eritrea, Ethiopia, France, Ghana, Indonesia, Iran, Iraq,Italy, Kenya, Kuwait, Lebanon, Malaysia, Mexico, Morocco, Nepal, Nigeria, Oman, Saudi Arabia, Somalia, South Africa, Sri Lanka, Sweden, Tunisia, Turkey, United Arab Emirates, United Kingdom, United States of America, Yemen.

**Abbreviations**: SD, Standard Deviation; CKD, Chronic Kidney Disease; CHF, Congestive Heart Failure; CVDs, Cardiovascular Diseases; AFib, Atrial Fibrillation; PVD, Peripheral Vascular Disease.

### Blood Pressure Control Among Patients with Hypertension Attending PHCC Centers in Qatar

Table 3 summarizes the prevalence of patients with controlled blood pressure over the years. Results are also stratified by age and certain comorbidities,

**Table 3:** Blood Pressure (BP) Control Assessment Using PHCC Hypertension Guidelines Across the Years

BP Measurement Date	Guideline Used for Assessment	Categories	Controlledn (%)	95% Confidence Interval	Total N of Patients
**2018**	**PHCC (2016)** **Adopted from JNC8 guideline (2014)**	<60 years hypertensive patients without diabetes or CKD (Target BP <150/90mmHg)	247(62.8%)	(58.1–67.6%)	393
		≥ 60 years hypertensive patients without diabetes or CKD (Target BP <140/90mmHg)	97 (61.8%)	(54.2–69.4%)	157
		Diabetic or CKD patients (Target BP <140/90mmHg)	882 (67.4%)	(64.8–69.9%)	1309
**2019**	**PHCC (2016)** **Adopted from JNC8 guideline (2014)**	<60 years hypertensive patients without diabetes or CKD (Target BP <150/90mmHg)	297 (64.3%)	(59.9–68.7%)	462
		≥ 60 years hypertensive patients without diabetes or CKD (Target BP <140/90mmHg)	99 (56.9%)	(49.5–64.2%)	174
		Diabetic or CKD patients (Target BP <140/90mmHg)	1002(71.5%)	(69.1–73.8%)	1402
**2020**	**PHCC (2020)** **Adopted from NICE guideline (2019)**	≥ 80 years regardless of their comorbidities (Target BP <150/90mmHg)	77 (81.9%)	(74.1–89.7%)	94
		<80 years regardless of their comorbidities (Target BP <140/90mmHg)	1152(63.9%)	(61.7–66.1%)	1803

**Abbreviations**: PHCC, Primary Health Care Corporation; JNC8, Eighth Joint National Committee; NICE, National Institute for Health and Care Excellence; CKD, Chronic Kidney Disease.

**Figure 1:**
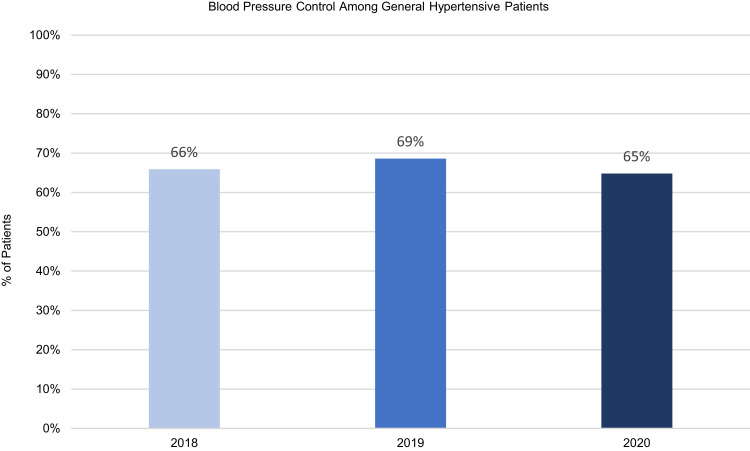
Blood Pressure Control Among General Hypertensive Population Regardless of Comorbidity (Total N=1859 in 2018, N=2038 in 2019, N=1897 in 2020).

**Figure 2:**
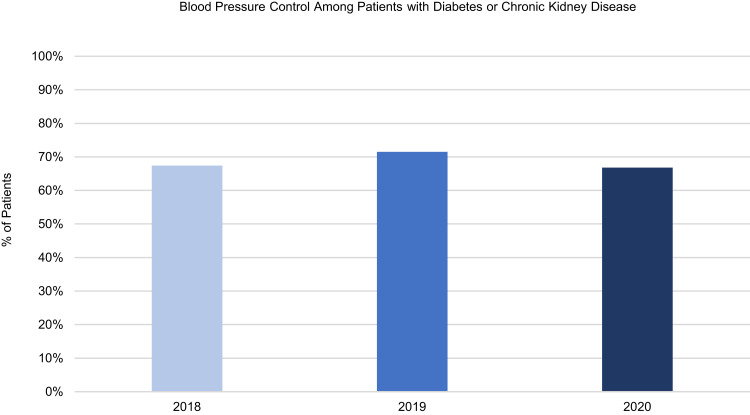
Blood Pressure Control Among Hypertensive Patients with Diabetes or Chronic Kidney Disease (Total N=1309 in 2018, N=1402 in 2019 N=1314 in 2020).

**Figure 3:**
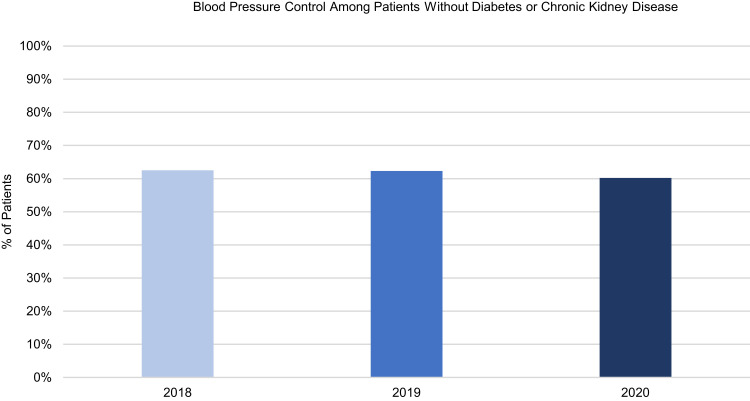
Blood Pressure Control Among Hypertensive Patients without Diabetes or Chronic Kidney Disease (Total N=550 in 2018, N= 636 in 2019 N=583 in 2020).

Additionally, Figures 1–3, based on data extracted from Cerner^®^, illustrate the percentage of patients achieving target blood pressure levels from 2018 to 2020. These figures categorize patients into three groups: the general population, patients with diabetes or chronic kidney disease (CKD), and those without diabetes or CKD. Over 60% of hypertensive patients attending PHCC centers had their blood pressure controlled from 2018 to 2021. Specifically, Figure 1 presents BP control for the general hypertensive population, Figure 2 depicts BP control among patients with diabetes or CKD, and Figure 3 shows BP control among patients without diabetes or CKD.

### Predictors of Blood Pressure Control Among Patients with Hypertension

#### Attending PHCC Centers in Qatar

The results of the univariable binary logistic and multivariable binary logistic regression are illustrated in Table 4. Each column refers to the results of one model. The findings from the univariable binary logistic regression analysis revealed that several factors were significantly associated with blood pressure (BP) control. Age, Qatari nationality, and comorbidities such as dyslipidemia, congestive heart failure (CHF), peripheral vascular disease (PVD), and atrial fibrillation (Afib) were positively associated with BP control. Conversely, male gender and diabetes were negatively associated with BP control.

**Table 4:** Univariable and Multivariable Binary Logistic Regression Models^a^

	Univariable Binary Logistic Regression (N=2185)				Multi-regression Model 1 (N=1897)			Multi-Regression Model 2 (N=1400)			Multi-Regression Model 3 (N=1036)	
	Unadjusted OR	95% CI	P-value	adjusted OR	95% CI	p-value	adjusted OR	95% CI	p-value	adjusted OR	95% CI	p-value
**Age**	1.008	(1.000-1.016)	0.043	0.998	(0.989-1.007)	0.636	1.002	(0.993-1.012)	0.615	1.007	(0.995-1.018)	0.264
**Males vs. females**	0.676	(0.560-0.817)	<0.001	0.736	(0. 602-0.900)	0.003	0.803	(0.637-1.011)	0.062	0.843	(0.644-1.103)	0.213
Qatari vs. Non-Qatari	1.540	(1.274-1.861)	<.001	1.278	(1.039-1.572)	0.020	1.308	(1.032-1.658)	0.027	1.481	(1.122-1.956)	0.006
**Nondiabetic vs. Diabetic**	1.383	(1.134-1.687)	0.001	1.127	(0.904-1.406)	0.288	1.145	(0.889-1.475)	0.294	1.206	(0.903-1.612)	0.205
Dyslipidemia vs. no dyslipidemia	1.552	(1.282-1.878)	<0.001	1.450	(1.181-1.782)	<0.001	1.346	(1.062-1.705)	0.014	1.448	(1.099-1.906)	0.008
**CKD vs. no CKD**	1.197	(0.890-1.610)	0.233	0.694	(0.503-0.956)	0.025	0.695	(0.489-0.986)	0.041	0.727	(0.470-1.125)	0.152
**IHD or MI vs. no IHD or MI**	0.405	(0.137-1.202)	0.104	1.984	(0.615-6.397)	0.252	1.613	(0.468-5.557)	0.449	1.281	(0.355-4.623)	0.705
CHF vs. no CHF	2.377	(1.446-3.907)	<0.001	1.982	(1.157-3.396)	0.013	1.994	(1.117-3.562)	0.020	2.417	(1.163-5.025)	0.018
**PVD vs. no PVD**	4.950	(1.145-21.397)	0.032	3.700	(0.804-17.041)	0.093	2.013	(0.402-10.068)	0.394	1.659	(0.310-8.868)	0.554
**AFib vs. no Afib**	2.541	(1.383-4.667)	0.003	2.144	(1.133-4.059)	0.019	1.957	(0.953-4.020)	0.067	1.901	(0.810-4.463)	0.140
**Arrhythmia vs. no arrhythmia**	0.674	(0.282-1.613)	0.376	1.181	(0.479-2.916)	0.718	1.726	(0.607-4.903)	0.306	1.725	(0.594-5.008)	0.316
**Last therapy recorded monotherapy vs. multi**	NA	NA	NA	NA	NA	NA	1.190	(0.939-1.510)	0.151	NA	NA	NA
**therapy 2020: Mono vs. multi**	NA	NA	NA	NA	NA	NA	NA	NA	NA	1.205	(0.892-1.630)	0.224

**Abbreviations**: ^a^OR, Odds ratio; CI, Confidence Interval; CKD, Chronic Kidney Disease; IHD or MI, Ischemic Heart Diseases or Myocardial Infarction; CHF, Congestive Heart Failure; PVD, Peripheral Vascular Disease; Afib, Atrial fibrillation; Mono, Monotherapy; Multi, Multiple drug therapy (dual, triple, quadruple, multiple drug therapy); NA, Not Applicable.

Age was identified as a statistically significant predictor of blood pressure (BP) control, with older patients demonstrating better BP control (Unadjusted OR = 1.008, 95% CI = 1.000–1.016, P-value = 0.043, Cox & Snell R^2^ = 0.002). Gender also played a significant role, with males having significantly lower odds of achieving controlled BP compared to females (Unadjusted OR = 0.676, 95% CI = 0.560–0.817, P-value < 0.001, Cox & Snell R^2^ = 0.009). Additionally, nationality was a significant predictor, with Qataris being more likely to achieve BP control compared to non-Qataris (Unadjusted OR = 1.540, 95% CI = 1.274–1.861, P-value < 0.001, Cox & Snell R^2^ = 0.011).

The combined effects of various determinants on BP control among hypertensive patients were analyzed using a multivariable regression model. The analysis revealed that these factors remained significant predictors: males were less likely to achieve BP control compared to females (adjusted OR = 0.736, 95% CI = 0.602–0.900, P-value = 0.003), while Qataris were more likely to have controlled BP compared to non-Qataris (adjusted OR = 1.278, 95% CI = 1.039–1.572, P-value = 0.020). Additionally, patients with dyslipidemia were more likely to maintain BP control (adjusted OR = 1.450, 95% CI = 1.181–1.782, P-value < 0.001). CKD emerged as a significant predictor for poorer BP control (adjusted OR = 0.694, 95% CI = 0.503–0.956, P-value = 0.025). Furthermore, patients with CHF and Atrial fibrillation were almost twice as likely to achieve BP control compared to those without CHF (adjusted OR = 1.982, 95% CI = 1.157–3.396, P-value = 0.013) and AFib (adjusted OR = 2.144, 95% CI = 1.133–4.059, P-value = 0.019), respectively.

In the second model, the most recent therapy taken by patients was added to the first model. Factors including nationality, dyslipidemia, CKD, and CHF remained associated with BP control. The third model was similar to the second model but included those who had therapy in 2020. Nationality, dyslipidemia, and CHF were the factors that remained associated with BP control. More details can be found in Table 4.

## Discussion

To our knowledge, this is one of the first studies to assess BP control status among hypertensive patients in primary care settings in Qatar and to investigate the various factors influencing BP control.

Despite the global trend of poor BP control, this study found that BP control in Qatar, while not optimal, is comparatively higher than in many other countries. The proportion of hypertensive patients with controlled BP ranged between 65% and 69% over the three years studied. In contrast, a 2017 study in Iran, the Occupied Palestinian Territory, the Kingdom of Saudi Arabia (KSA), and the United Arab Emirates (UAE) reported that only 19% of hypertensive patients had controlled BP, with 47% receiving treatment.^35^ Similarly, a 2021 systematic review and meta-analysis of 178 studies in the MENA region also found a 19% BP control rate.^15^ In Arab countries, BP control rates varied significantly from 12% in Morocco and 13% in Tunisia to 57% in Algeria, 54% in Lebanon, and 67% in Kuwait.^16^ Suboptimal BP control was also observed in neighboring Gulf countries, with 38% in the UAE and 51.3% in primary care settings in KSA.^36^,^37^

The higher percentage of BP control in our study may be attributed to the focus on patients actively receiving antihypertensive treatment. Many studies in the literature assess prevalence, awareness, treatment, and control rates among all hypertensive patients, which may not accurately reflect control rates among those undergoing treatment. For example, in Korea, 46.2% of hypertensive patients had controlled BP, but this rate increased to 72.0% among those receiving treatment.^38^ Similarly, in Bangladesh, BP control rates rose from 12.7% to 43.6% when only treated patients were considered.^39^

Qatar’s status as a high-income country, with one of the highest gross domestic product (GDP) per capita rates, could also contribute to the higher BP control rates observed in this study.^40^ A systematic review indicated that high-income countries achieve BP control at rates four times higher than low- and middle-income countries.^41^ Our findings align with these trends, as comparable BP control rates have been reported in other high-income countries: 59.4% in Switzerland, and 64.6% in Canada.^42–44^ A 2022 cross-sectional survey using data from the National Health and Nutrition Examination Survey in the US revealed similar results, with BP control rates among patients on antihypertensives being 69.9% (2009–2012), 69.3% (2013–2016), and 67.7% (2017–2020).^45^

Global disparities in BP control are often due to challenges faced by low- and middle-income countries, such as limited access to healthcare, poor health literacy, non-adherence to guidelines, expensive medications, and healthcare provider burden.^46^ In contrast, Qatar offers exceptional access to healthcare, with PHCCs providing free services for local citizens and subsidized costs for non-Qatari residents, who only pay 20% of medication costs, often covered by insurance.^47^ Qatar’s healthcare system is committed to achieving the Qatar National Vision 2030 and has made significant investments in improving care quality, particularly for cardiovascular diseases, including hypertension.^48^ These investments may be associated with the moderately high BP control rates observed in this study in spite of the observational design of the study may impede a causal inference.^49^

Although BP control in Qatar is relatively higher than in many other countries, it remains suboptimal, with more than one-third of the study sample having uncontrolled BP. Non-adherence to medications is a likely contributing factor.^50^ While no studies have specifically investigated non-adherence to antihypertensive therapy in Qatar, a study on patients with uncontrolled diabetes, where 75% had co-existing hypertension, found that approximately 73% of patients self-reported non-adherence.^51^ Factors such as lack of family support, patient beliefs and behaviors, and forgetfulness were associated with poor adherence.^51^

Another contributing factor for the BP control rates in Qatar could be unhealthy lifestyle choices, such as lack of physical activity and poor dietary habits. Over 63% of Qatar’s population under 65 does not engage in recreational physical activities,^52^,^53^ and the National STEPwise survey (STEPS) revealed that 70% of the population is overweight, with 40% classified as obese.^53^ The country’s economic growth in Qatar has led to a shift from a traditional Mediterranean-style diet to unhealthy fast foods high in sugar and carbohydrates.^54^ Implementing interventions targeting regular exercise and healthier eating habits would be a potential promising strategy for managing hypertension and improving overall health in this population. Nevertheless, due to the observational design of the study and the distinctive sociodemographic characteristics of Qatar’s population, these clinical implications should be considered carefully and may have limited applicability in other healthcare settings.^22^ While 89.3% of physicians in Qatar advise their patients on lifestyle modifications, further research is needed to explore barriers to changing physical activity and dietary habits and their impact on BP control.^55^ Additionally, investigating disparities among various racial and ethnic groups, which make up 88.4% of Qatar’s population,^56^,^57^ is essential, as BP control may vary due to differences in health literacy, income, awareness, and lifestyle choices.^58^

An interesting finding of the study was that patients with diabetes or CKD exhibited better BP control, representing 75.4% of the treated hypertensive patients. The percentage of patients achieving a target BP of <140/90 mmHg was 67.4% in 2018, 71.5% in 2019, and 66.8% in 2020. These results are notable, given the typically lower BP control rates reported in patients with diabetes and CKD. For instance, a meta-analysis across 20 countries found an overall BP control rate of 29% among patients with hypertension and type 2 diabetes, with significant regional variation.^59^ In Spain, only 17.5% of diabetic patients met a tighter BP target of <130/80 mmHg, compared to 36.9% at <140/85 mmHg.^60^ Similarly, in Nigeria, just 17.1% of patients with both hypertension and type 2 diabetes had controlled BP (<130/80 mmHg).^61^ The Veterans Affairs Health Care System reported a 77.5% BP control rate among diabetic patients, but this dropped to 39.8% with optimal glycemic control^62^ In Malaysia, 47.2% of patients with both conditions achieved controlled BP within tight ranges.^63^ The higher BP control rates in our study may be due to using the standard target of <140/90 mmHg from the JNC8 guidelines, rather than the stricter targets used elsewhere. Additionally, better BP control in the CKD population in our study, compared to other studies,^64–67^ could be attributed to greater disease awareness and more consistent antihypertensive medication use among patients managing comorbidities such as diabetes and CKD; However, owing to the retrospective nature of the study, medication adherence was not assessed and this explanation could be hypothetical.^68^ This study used univariate and multivariate binary logistic regression analyses to identify factors influencing BP control among hypertensive patients. While advanced age initially appeared significant in univariate analysis, its significance diminished in multivariate models, indicating that age alone may not directly predict BP control. This might be because older patients often have comorbidities that contribute to better BP control. Similar findings were reported in Iran, where age was not a significant predictor in multiple logistic regression analyses across different age groups.^42^ The literature shows mixed results: some studies suggest older patients achieve better BP control due to improved medication adherence and disease awareness, while others link older age (≥ 60 years) to poorer BP control.^35^,^37–39^,^69–72^ Nationality remained a significant predictor in both univariate and multivariate analyses, with Qataris more likely to have controlled BP than non-Qataris possibly due to differences in baseline BP, lifestyle choices, and social determinants of health.^58^

Gender was a significant predictor, with males less likely to achieve BP targets than females, as shown in both univariate analysis and the first multivariate model. Previous studies offer mixed findings on gender and BP control—some report higher rates of uncontrolled BP in females^16^,^73^,^74^ while others align with our results, showing better BP control in females.^15^,^35^,^38^,^69^,^75^ This may be because women tend to adhere more closely to treatment regimens^76^ with research indicating higher adherence to antihypertensive therapy among women compared to men.^77^ Gender disparities in BP control may also be influenced by factors such as smoking status, physical activity levels, dietary habits, and underlying physiological differences.^78^,^79^

The study models revealed that patients with certain comorbidities, such as dyslipidemia, CHF, Afib, and PVD, had better BP control, while those with diabetes and CKD had poorer BP control. Similar patterns were observed in hypertensive patients from general practices in Denmark^80^ and in a UK cohort study across 674 general practices where comorbidities were linked to lower BP readings.^81^ Patients with hypertension and dyslipidemia often achieve better BP control due to their increased CVD risk,^82^ leading healthcare professionals to provide more intensive care.^83^ Additionally, statin therapy and lifestyle modifications, such as dietary changes and improved physical activity, contribute to better BP control in these patients.^82^,^84^

Improved BP control in patients with Afib and CHF may be due to the increased use of antihypertensive medications like beta-blockers, Angiotensin Converting Enzyme (ACE) inhibitors, and Angiotensin Receptor Blockers (ARBs), which also lower BP levels.^80^,^81^,^85^,^86^ This pattern is consistent with prescribing trends reported in primary care settings in Qatar, where these drug classes were among the most commonly prescribed antihypertensive agents.^26^ This could also explain why patients with PVD had four times better BP control, as ACE inhibitors are commonly prescribed to high-risk cardiovascular patients, enhancing peripheral perfusion and lowering BP^87^ However, this significance was only observed in univariate analyses, losing significance when accounting for other confounding variables.

### Strengths and Limitations

The study had several strengths. Firstly, it utilized a large sample size of hypertensive patients, enhancing the robustness and generalizability of the findings. Conducting the study within a primary care setting was crucial, as it represents the first point of contact for many patients.^88^ The use of a standardized data collection document ensured internal validity and reproducibility,^89^ while precise variable definitions reduced the risk of misclassification bias.^90^ Moreover, the use of multivariate analyses to adjust for possible confounders was a key strength.^91^

However, the study faced some challenges, leading to certain limitations. Extracting all patient data and records from Cerner^®^ was difficult, as this task was assigned to the IT team, limiting data accessibility. To address this, regular meetings were held with the IT team to optimize data extraction. Additionally, the lack of records before Cerner^®^ implementation in 2017 and the absence of documented BP measurements for patients treated in 2017 and 2021 limited the data’s comprehensiveness. Moreover, the retrospective observational design of this study does not allow conclusions about cause and effect. Therefore, the relationships observed between patient characteristics and blood pressure control should be viewed as associations that may help generate hypotheses rather than confirm causality. In addition, several factors known to affect blood pressure control were not available in the reviewed database and may have contributed to residual confounding. Most notably, medication adherence, which plays a central role in the effectiveness of antihypertensive treatment,^92^ was not measured. Key lifestyle factors were also unavailable, including physical activity, dietary habits and intake, alcohol and tobacco use, all of which are well-established contributors to blood pressure outcomes.^21^,^22^ Socioeconomic variables such as educational level, employment status, and health literacy were also not captured. This may limit the ability of the models to fully explain the findings and may have left some confounding factors unaccounted for. The time it took for hypertensive patients to achieve blood pressure control was not captured, yet this was not the objective of the study.

Future studies should include validated tools to prospectively measure these variables in order to have an overall understanding of all potential determinants of BP control in this population. Additionally, developing and testing interventions to overcome barriers to BP control is crucial to optimize the suboptimal levels observed among some patients. Multi-center studies are also recommended to improve the generalizability of the findings.

## Conclusion

In conclusion, this retrospective observational study found that over half of hypertensive patients in Qatar’s primary care setting achieved controlled BP. Through regression models, significant predictors of BP control were identified, including Qatari nationality, dyslipidemia, and CHF. These findings suggest that Qatari patients and those with CHF or dyslipidemia are more likely to achieve controlled BP. Nevertheless, given the retrospective design of this study, these associations should not be considered causal relationships, and the generalizability of the study findings beyond Qatar’s primary care setting requires caution.

On the other hand, the study showed that many hypertensive patients continue to have uncontrolled blood pressure, highlighting the need to address barriers to optimal BP control. Future prospective studies are needed to confirm the observed predictors. These studies should also include validated assessments of medication adherence and lifestyle factors that could affect blood pressure control. This would help design targeted strategies to improve blood pressure control in this population.

## Data Availability

Data is available and can be provided by the corresponding author on a reasonable request.
